# An atypical multiple autoimmune syndrome: A case report including myocarditis

**DOI:** 10.1016/j.radcr.2025.02.067

**Published:** 2025-03-18

**Authors:** Ikram Tahani, Fadoua Achour, Noha El Ouafi, Nabila Ismaili

**Affiliations:** a Laboratory of Epidemiology, Clinical Research and Public Health, Faculty of Medicine and Pharmacy, Oujda, Morocco; b Department of Cardiology, Mohammed VI University Hospital of Oujda, Oujda, Morocco

**Keywords:** Multiple Autoimmune syndrome, Acute myocarditis, Rheumatoid arthritis, Gougerot-Sjögren's syndrome

## Abstract

The co-occurrence of autoimmune diseases has been epidemiologically studied and has contributed to our understanding of autoimmunity. The underlying mechanisms of this syndrome remain elusive; however, its prevalence may be higher than currently documented. The co-occurrence of at least 3 autoimmune diseases in a single patient has been defined as multiple autoimmune syndrome (MAS). This syndrome can be classified into 3 categories based on the prevalence of their associations with one another: type 1, type 2, and type 3. Myocarditis is classified as type 3, and typically does not present with connective tissue involvement. This report presents the case of a young female with known autoimmune disorders: rheumatoid arthritis (RA)and secondary Sjögren's syndrome (GSJ). The patient presented with MRI-confirmed myocarditis, likely of autoimmune origin, combining the 3 conditions in an atypical presentation. An additional noteworthy aspect of this case is the uncommon occurrence of myocarditis in rheumatoid arthritis, and even more so in Sjögren's syndromes.

## Introduction

Myocarditis, a major cause of heart disease in young patients, and a common precursor to heart failure and dilated cardiomyopathy, can occur in multiple autoimmune diseases. When 3 or more of these diseases coexist, the condition is referred to as multiple autoimmune syndrome (MAS) [[1], [2], [3]]. The coexistence of 5 autoimmune diseases is extremely rare. Familial, genetic, infectious, immunological, and psychological factors have been implicated in MAS development [4,5]. Based on this observation and supported by the literature, we aimed to highlight the unique features of myocarditis in the context of rheumatoid arthritis (RA) and Sjögren's syndromes, particularly in the setting of an atypical MAS presentation.

## Case presentation

Our case involved a 46-year-old female with a history of erosive, seropositive rheumatoid arthritis (RA) associated with secondary Gougerot-Sjögren's syndrome (GSJ) for the past 4 years. She was treated with 20 mg methotrexate weekly and had a history of corticosteroid-induced adrenal insufficiency, for which she was administered 20 mg hydrocortisone daily. Additionally, she received adjuvant therapy including folic acid and vitamin D, although her medication compliance was poor.

**Fig. 1:**
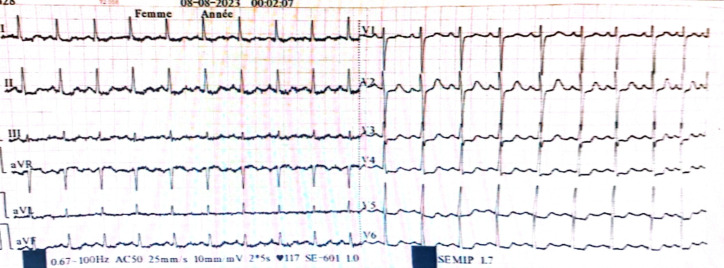
Electrocardiogram (EKG) performed at the regional hospital showing a regular sinus rhythm with an anteroseptoapical and low inferolateral ST-segment shift.

**Fig. 2:**
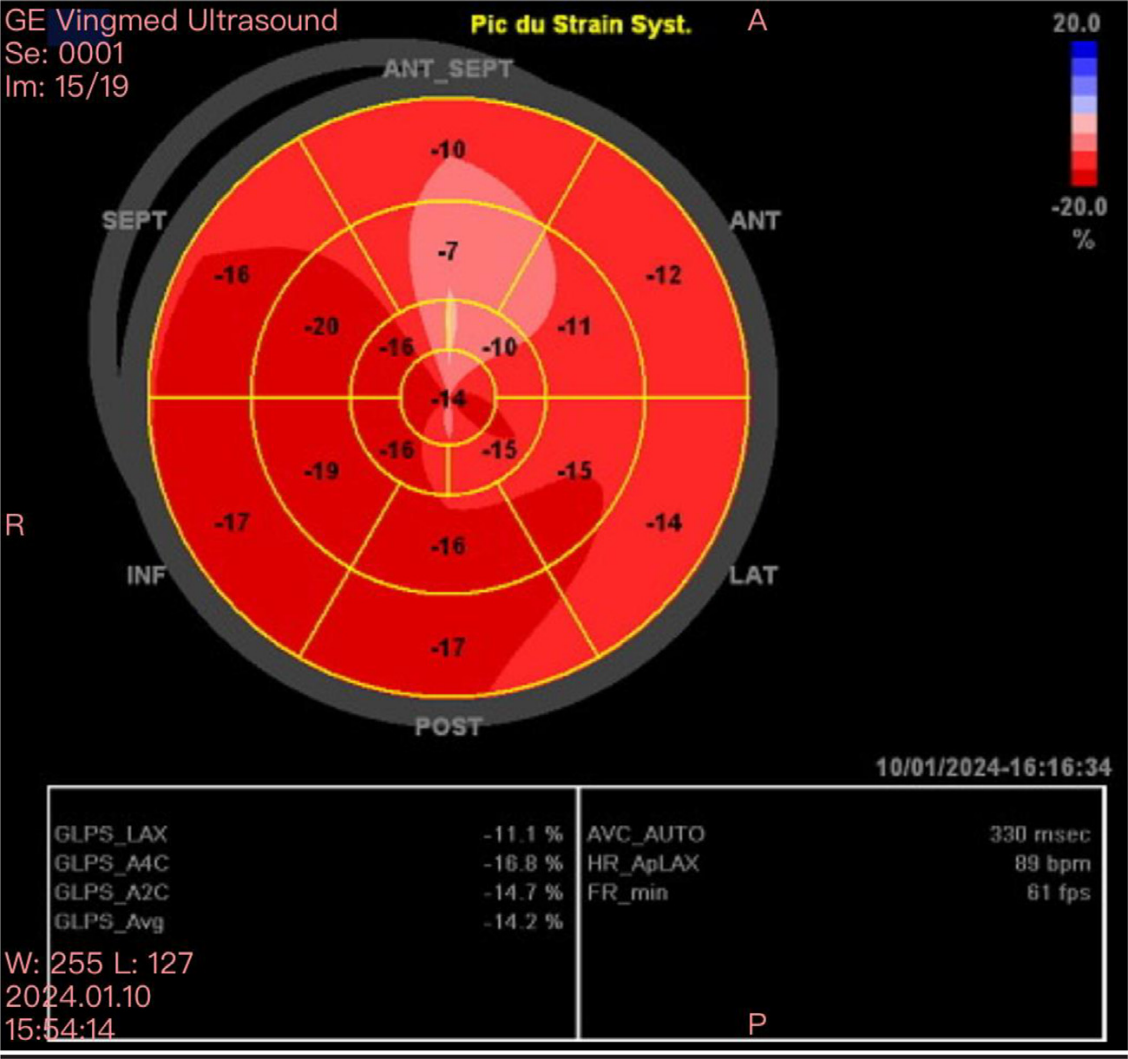
Echocardiography (ETT) showing global longitudinal strain reduced to −14%, at the expense of lateral and anterior walls.

She presented to the emergency department of our training center with acute chest pain dating back to the same day, occurring in the context of chronic fever, without symptoms of flu-like syndrome. Initial clinical examination revealed a conscious patient with blood pressure of 120/60 mmHg respectively for systolic and diastolic blood pressure, heart rate of 80 bpm, and unremarkable cardiovascular examination. However, joint examination revealed swelling of the left wrist with preserved motor function and sensation. Electrocardiogram (EKG) showed regular sinus rhythm with an anteroseptoapical and low lateral ST-segment shift (Fig. 1). Echocardiography revealed a nondilated left ventricle (LV) with lateral and anterior hypokinesis. Left ventricular ejection fraction (LVEF) was preserved at 59%, but global longitudinal strain was reduced to −14%, specifically affecting the same walls (Fig. 2).

**Fig. 3:**
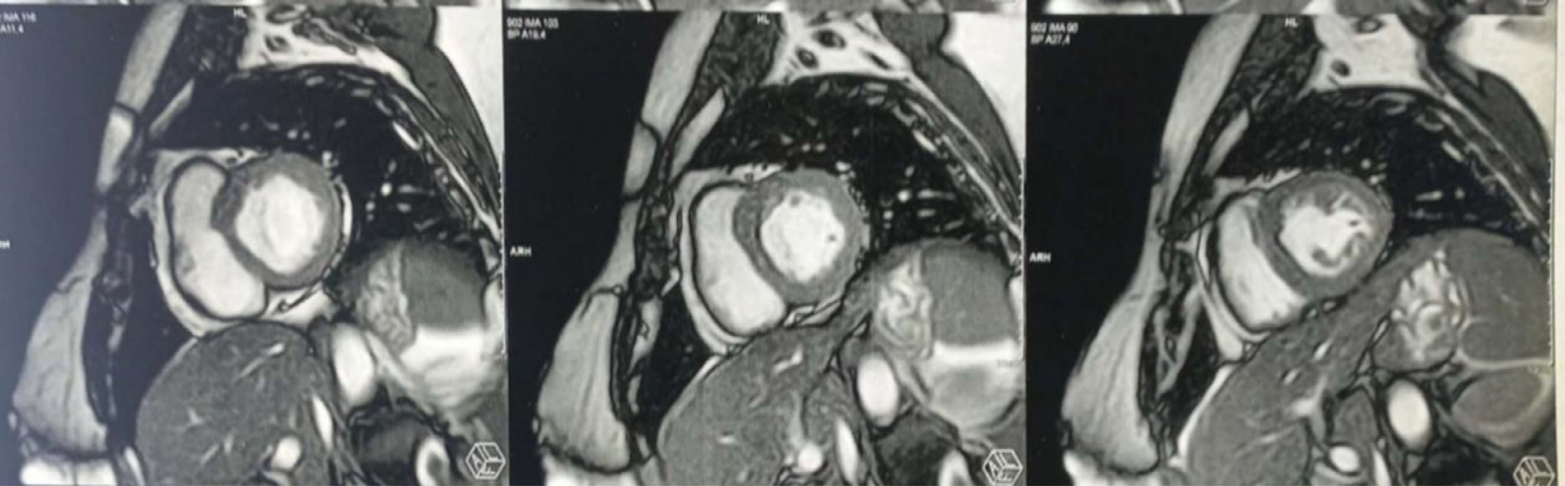
T2 short-axis cardiac MRI sequence demonstrating significant edema in the lateral wall.

**Fig. 4:**
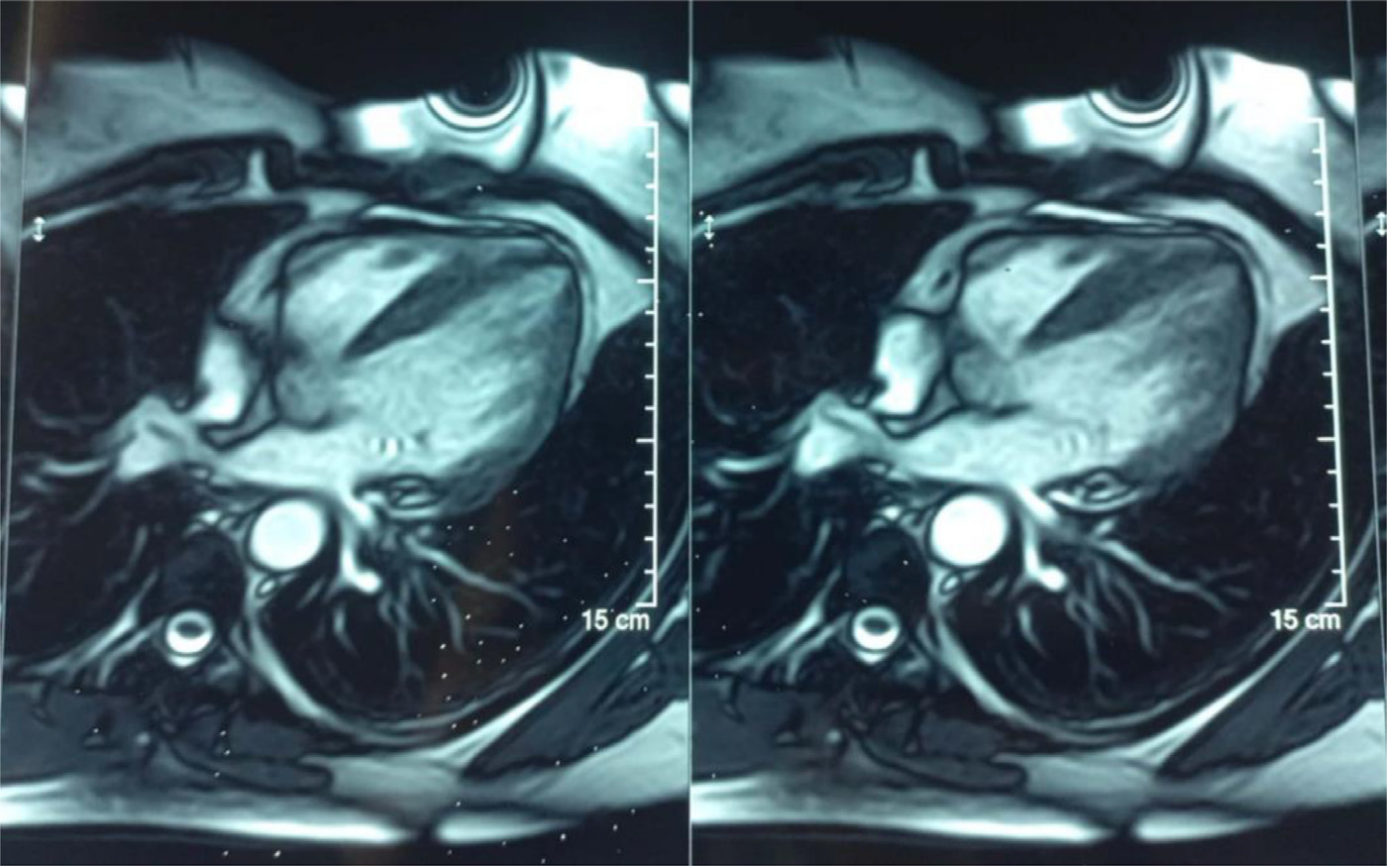
Cardiac MRI 4-chamber sequence showing late subepicardial enhancement in the middle and apical segments of the lateral wall.

There was no significant valve disease, and the pericardium appeared to be dry. The coronary network was angiographically normal. Cardiac MRI confirmed the diagnosis, showing all the Lake Louise criteria's with T1 and T2 signal abnormalities; myocardial edema (Fig. 3), early and late gadolinium enhancement of epicardial localization (Fig. 4), for our patient, the abnormalities were localized to the middle and apical segments of the lateral wall.

Biological data revealed a markedly elevated troponin level of 1090 ng/ml (normal range: <26 ng/ml). The inflammatory markers were mildly abnormal, likely due to hydrocortisone therapy, with a C-reactive protein (CRP) level of 3.29 mg/l (normal range: <6 mg/l), white blood cell count of 10,570 cells/μl (normal range: 4,000-10,000 cells/μl), and fibrinogen level of 3 g/l (normal range: 2-4 g/l). Despite the absence of flu-like symptoms and chronic fever, nasopharyngeal testing for COVID-19, respiratory syncytial virus, and influenza were performed all returned negative. Viral serology indicated immunity against cytomegalovirus (CMV), suggesting a possible autoimmune etiology for myocarditis.

**Fig. 5:**
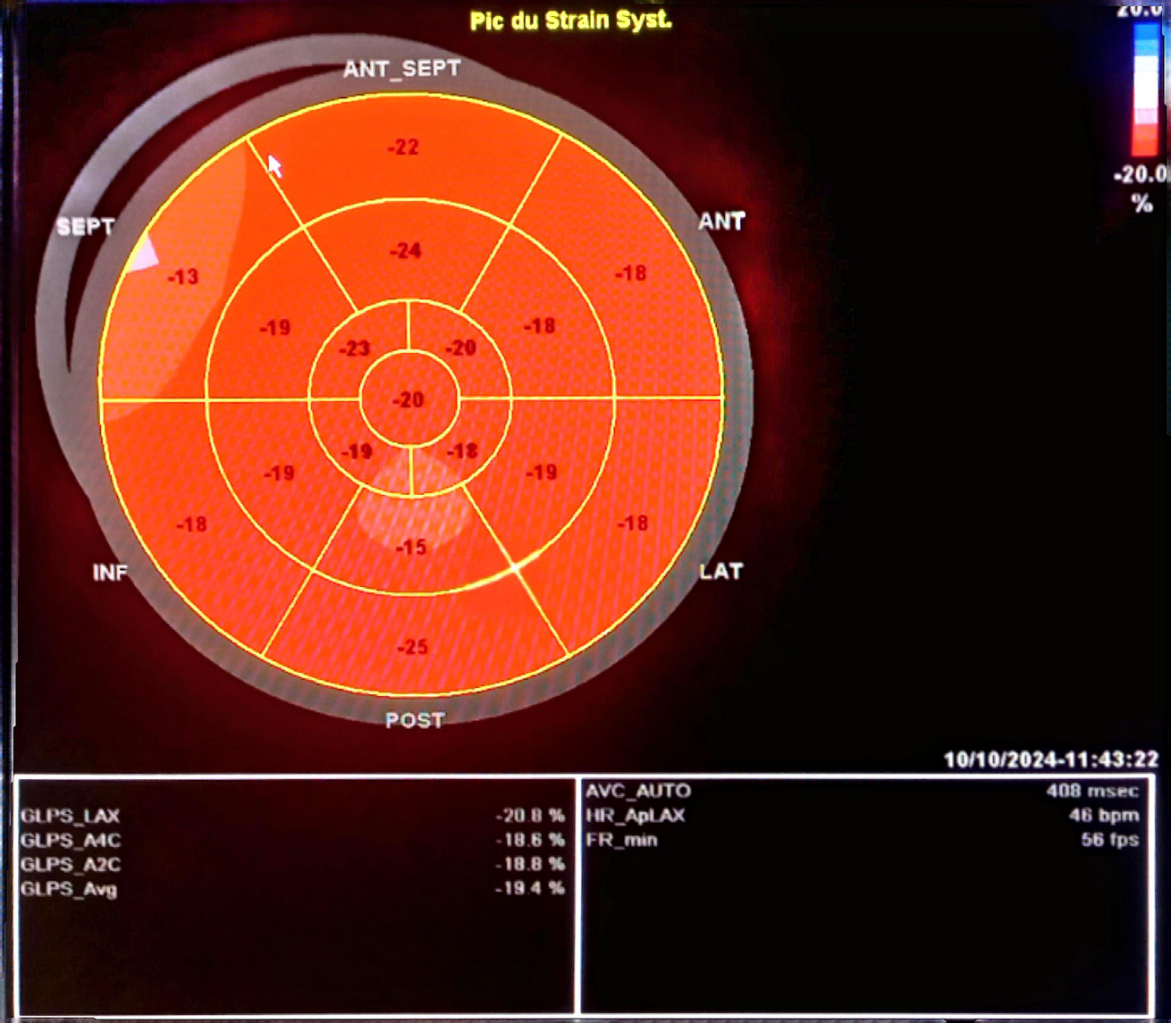
Echocardiography (ETT) showing improvement in global and segmental systolic function with a global longitudinal strain of −19.4%.

The patient was admitted to the cardiac intensive care unit where no rhythm disturbances were observed. A favorable clinical progression was noted, with the patient demonstrating good tolerance to the initiation of both the angiotensin-converting enzyme inhibitor (ramipril 5 mg) and the beta-blocker (Bisoprolol 2.5 mg). Weekly methotrexate doses were maintained, with a strong emphasis on ensuring therapeutic compliance. The patient was discharged after a 3-day stay. During the hospitalization, follow-up echocardiography revealed a left ventricule of normal size and a significant improvement in global and segmental systolic function with an ejection fraction of 58% and a global longitudinal strain of −19.4% (Fig. 5).

## Discussion

MAS is classified into 3 groups that correspond with the prevalence of their associations with one another (1);

1. Type 1 MAS includes myasthenia gravis, thymoma, polymyositis and myocarditis.
2. Type 2 MAS includes Sjögren's syndrome, rheumatoid arthritis (RA), primary biliary cirrhosis (PBC), scleroderma, and autoimmune thyroid disease.
3. Type 3 MAS groups together autoimmune thyroid disease, myasthenia gravis and/or thymoma, Sjögren's syndrome, pernicious anemia, idiopathic thrombopenic purpura (ITP), Addison's disease, type 1 diabetes mellitus, vitiligo, autoimmune hemolytic anemia (AIHA), Systemic Lupus Erythematosus (SLE), and dermatitis herpetiformis.

Autoimmune disorders affect approximately 3%-5% of the general population (6) and are even rarer when they come together. Approximately 25% of patients with autoimmune diseases tend to develop additional autoimmune conditions [7]. The occurrence of MAS suggests the presence of environmental triggers and genetic susceptibility [1]. Cytomegalovirus (CMV), for instance is a ubiquitous virus belonging to the *Herpesviridae* family [6] and is involved in pathogenesis. Its ability to induce RA has not been clearly elucidated, as is the case with Epstein-Barr virus (EBV), but its capacity to amplify and maintain the dysimmune response has been well and truly proven [1]. Three main mechanisms have been described [6]: autoantibody production, viral epitopes, structurally similar to self-ones, can induce the activation of both T- and B-cells through their presentation by APCs, enhanced cytokine-mediated release by T lymphocytes, and vascular damage .In this regard, it is important to point out that RA patients tend to display expansion of a particular subset of T-cells CD4+ lacking the costimulatory molecule CD28, which causes major coronary damage. Intriguingly, human CMV infection is a major trigger of CD4+CD28−*T*-cell expansion [8]. Interestingly, anti-CMV IGGs were also observed in our patient. To our knowledge, the association of rheumatoid arthritis, secondary Gougerot-Sjögren syndrome and myocarditis has never been previously described, reflecting the authenticity of our case.

Autoimmune myocarditis (AM) is primarily mediated by CD4+ *T* cells, with Th1 and Th17 cell infiltrates being identified in the hearts of murine models [9]. Endomyocardial biopsy remains the gold standard for diagnostic confirmation [3], although it is not routinely performed due to its low sensitivity and potential complications. Myocarditis can accompany various autoimmune and/or auto-inflammatory diseases, including sarcoidosis, rheumatoid arthritis (RA), Behçet's disease, eosinophilic granulomatosis with polyangiitis, myositis, and systemic lupus erythematosus [3]. Our patient was under care for rheumatoid arthritis and secondarily diagnosed with Gougerot-Sjögren's Syndrome (GSJ), both can induce autoimmune myocarditis, which is the suspected diagnosis for our patient.

Rheumatoid arthritis (RA) is a T-cell-driven autoimmune disorder characterized by autoantibody production, which primarily affects the synovial lining of joints, leading to destructive synovitis, progressive disability, and potentially premature death due to extra-articular manifestations, including cardiovascular (CV) or neurovascular events [10]. Among the most common cardiovascular manifestations of RA are ischemic heart disease and congestive heart failure (CHF) [11]. Myocarditis, a condition associated with CHF, has been reported in 11%-50% of postmortem analyses of RA patients [12,13]. In contrast, other cardiac complications, such as pericarditis, endocarditis, cardiac tamponade, and long-term development of amyloidosis are relatively rare [11]. The proinflammatory cytokines primarily involved in the nonischemic cardiac damage observed in RA include tumor necrosis factor-alpha (TNF-α) and interleukin-1 (IL-1) [14]. Elevated levels of antibodies against citrullinated protein antigens (ACPAs) have also been implicated in cardiac damage [15]. While ischemic damage in RA is primarily associated with the pathogenic effects of lipoprotein abnormalities, endothelial dysfunction, and inflammation, these factors play a direct role in the development of atherosclerosis.

Sjögren's syndrome (SS) is one of the most frequent rheumatic diseases, characterized by lymphocytic infiltrates in the lacrimal and salivary glands, causing keratoconjunctivitis sicca and xerostomia, which are the main clinical manifestations of the disease [16]. This latter can be primary (pSS) or in most cases secondary to other connective tissue disorders particularly Systemic Lupus Erythematosus (SLE) and rheumatoid arthritis (RA) . Studies conducted by Brito-Zerón et al. [17] indicated that cardiovascular diseases are the leading cause of death in the largest cohort of patients with primary SS, accounting for 30% of fatalities. In a similar context, Vaudo et al. (2005) [18] observed a higher prevalence of subclinical atherosclerosis in 37 female patients with SS, as assessed through femoral and carotid ultrasonography. Although severe complications such as acute pericarditis and myocarditis have rarely been reported, the most well-known cardiac complication associated with SS is congenital heart block, particularly in patients with positive anti-Ro/SSA antibodies [16].

To the best of our knowledge, there have been no specific MRI signs of rheumatoid myocarditis reported [19]. However, these patients tend to have higher T1, T2, and extracellular volume fraction (ECV) values than controls, along with a modest prevalence of late gadolinium enhancement (LGE) at 18% [20]. These abnormalities may also be observed in other autoimmune rheumatic diseases (AIRD), such as systemic lupus erythematosus (SLE), systemic sclerosis (SSc), and inflammatory myopathies [20]. In addition to its diagnostic and prognostic value, cardiac MRI is considered the test of choice for monitoring cardiotoxicity in patients undergoing long-term treatment with disease-modifying antirheumatic drugs (DMARDs) [21].

While myocardial biopsy can be useful for distinguishing between autoimmune myocarditis and viral myocarditis by identifying the causative agent in the case of viral infections, it is less informative when inflammatory infiltrates are present without identifiable pathogens (such as lymphocytic or other forms of infiltration), as these can be found in all forms of myocarditis, including viral, autoimmune, and toxic etiologies [22]. Myocardial biopsy is typically reserved for cases of fulminant myocarditis that do not improve after a few days or when a nonlymphocytic cause is suspected, such as in cases of ventricular arrhythmias or high-grade conduction disorders indicative of giant cell myocarditis (GCM) [22].

Interstitial and granulomatous forms of myocarditis have been the most frequently described in the course of RA [23], with greater specificity for the granulomatous form, although giant cell myocarditis; a form assumed to have an immunogocial mechanism with worse prognosis, have also been reported [24,25] Notably, 19% of GCM cases were found to have concomitant autoimmune disorders [25]. Conditions such as autoimmune thyroid disease, RA, myasthenia gravis, aortitis, vitiligo vulgaris, pernicious anemia, inflammatory bowel disease, and orbital myositis have all been associated with GCM [25]. In this context, aggressive immunosuppressive therapy appears to improve the prognosis of affected patients [25].

Biologically, autoantibodies targeting heart muscle components, such as cardiac myosin, are not exclusive to patients with myocarditis but are also detected in individuals with other cardiovascular diseases [26]. Eriksson et al. reported the presence of autoantibodies against cardiac troponin I (cTnI) and cardiac troponin T (cTnT) in patients with dilated cardiomyopathy (DCM) or ischemic cardiomyopathy (ICM) [26]. Furthermore, the detection of nuclear antibodies would not contribute significantly to the diagnosis of autoimmune myocarditis in our patient, as she was experiencing a relapse of her rheumatoid disease. It is also important to note that viral myocarditis can evolve into autoimmune myocarditis due to the production of antimyosin autoantibodies following the formation of monocytic infiltrates [27].

The guideline-based therapy for patients with acute myocarditis is supportive therapy for left ventricular dysfunction, a treatment approach that was adopted in the management of our patient. She was put on a beta-blocker and angiotensin-converting enzyme (ACE) inhibitor, as these medications not only mitigate heart failure but also provide significant anti-inflammatory effects [27]. Engman et al. compared the use of angiotensin-converting-enzyme inhibition and angiotensin II receptor blockade in experimental autoimmune myocarditis and found that both drugs significantly reduce inflammation, necrosis, and fibrosis in myosin-immunized mice [27].

MTX(methotrexate), an antifolate antimetabolite, which considered to be a powerful agent targeting autoimmune diseases and cancer, is classically associated with improved endothelial function, slower atherosclerosis progression, decreased risk of major cardiovascular adverse events, and benefits on cardiovascular survival [28]. These benefits are thought to be due to its antiproliferative, immunosuppressive, anti-inflammatory, and antiatherogenic effects. Cardiotoxicity, which may lead to myocarditis, occurs mainly from polypharmacy, overdosage, and long pharmacotherapy with conventional disease-modifying antirheumatic drugs (DMARD), renal impairment, folate deficiency and biologic agents such as Tumor Necrosis Factor (TNF)-α blockers and IL-6 receptor Inhibitors [20]. In such cases, the use of leucovorin (or folinic acid) at a dose of 100 mg/m² every 6 hours for 24 hours is recommended to mitigate potential toxicity [28].

Our patient did not exhibit renal insufficiency and was not on any treatments that would interfere with methotrexate. She was therefore continued on methotrexate, with a strong emphasis on patient compliance, as monitored by our rheumatology colleagues.

## Conclusion

Cardiovascular disease has been well-documented in various autoimmune disorders. Rheumatoid arthritis (RA), for instance, is recognized as a significant cardiovascular risk factor and has recently been shown to contribute to myocardial insufficiency. In the case of Gougerot-Sjögren syndrome, cardiovascular damage has been extensively confirmed in numerous studies, particularly in the primary form of the disease. According to the European Society of Cardiology (ESC) recommendations on cardiovascular risk prevention, a comprehensive assessment of cardiovascular risk is essential in patients with chronic inflammatory diseases, including RA. Myocarditis, in particular, presents one of the most challenging diagnoses in this context. The occurrence of myocarditis during the course of an autoimmune disease creates a complex clinical scenario, necessitating further research to better guide management strategies. Early recognition and timely treatment are critical to prevent potentially fatal outcomes associated with autoimmune myocarditis.

## Patient consent

I assure that the patient has given his written informed consent for the relating to the subject matter above (“An atypical multiple autoimmune syndrome: a case report including myocarditis”) to appear in a journal article, or to be used for the purpose of a thesis or presentation.
